# Manganese Superoxide Dismutase: Structure, Function, and Implications in Human Disease

**DOI:** 10.3390/antiox14070848

**Published:** 2025-07-10

**Authors:** Jovan Grujicic, Antiño R. Allen

**Affiliations:** 1 Division of Radiation Health, University of Arkansas for Medical Sciences, Little Rock, AR 72205, USA; jgrujicic@uams.edu; 2 Department of Pharmacology and Toxicology, University of Arkansas for Medical Sciences, Little Rock, AR 72205, USA; 3 Department of Pharmaceutical Sciences, University of Arkansas for Medical Sciences, Little Rock, AR 72205, USA; 4 Neurobiology & Developmental Sciences, University of Arkansas for Medical Sciences, Little Rock, AR 72205, USA

**Keywords:** manganese superoxide dismutase (MnSOD), oxidative stress, reactive oxygen species (ROS), mitochondria-targeted antioxidants

## Abstract

Manganese superoxide dismutase (MnSOD) is a vital mitochondrial antioxidant enzyme that preserves cellular integrity by catalyzing the dismutation of superoxide radicals into hydrogen peroxide. Its central role in maintaining redox homeostasis has positioned it as a key target in biomedical research. This review provides an in-depth examination of MnSOD’s structural and functional properties, regulatory mechanisms, and its involvement in the pathogenesis of various human diseases.

## 1. Introduction

Mitochondria are multifunctional organelles essential to processes such as ATP generation, calcium homeostasis, metabolite biosynthesis, and apoptosis regulation [1]. Among these, ATP production via oxidative phosphorylation is perhaps the most vital for life; however, it also results in reactive oxygen species (ROS) formation, primarily as superoxide anions (O_2_^•−^) due to electron leakage from the electron transport chain [2]. While physiological ROS levels play signaling roles, their excessive accumulation disrupts the redox balance and damages biomolecules, contributing to various diseases [1].

Superoxide dismutases (SODs) are metalloenzymes that catalyze the dismutation of O_2_^•−^ into oxygen and hydrogen peroxide (H_2_O_2_), preventing oxidative damage [3]. Based on their metal cofactors, SODs are classified into copper zinc SOD (SOD1), iron SOD (SOD3), manganese (Mn)SOD, and nickel SOD isoforms [4]. These enzymes are compartmentalized for optimal function: SOD1 is cytosolic and enriched in tissues like liver and kidney; SOD3 is extracellular in lower organisms but also found in chloroplasts [5,6]. In humans, SOD3 is largely confined to extracellular sites with limited expression.

**Figure 1:**
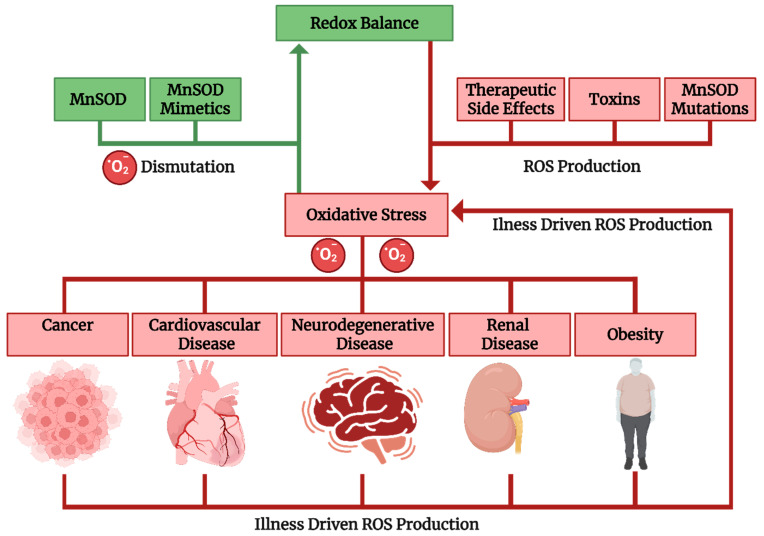
MnSOD and the maintenance of mitochondrial redox homeostasis.

MnSOD, localized in the mitochondrial matrix, is the primary antioxidant defense within mitochondria. When redox homeostasis is disturbed (e.g., genetic mutations, environmental toxins, or therapeutic side effects), oxidative stress develops. This stress contributes to the onset and progression of diseases such as cancer, cardiovascular disease, renal and neurodegenerative disorders, and obesity. These conditions can further enhance ROS production, forming a positive feedback loop that amplifies oxidative damage (Figure 1). Therefore, MnSOD is especially important in energy-demanding organs such as the heart, brain, and liver, where mitochondrial ROS production is highest [7].

Due to their essential role in oxidative homeostasis, SODs have emerged as key targets in research on inflammation, cancer, aging, and metabolic dysfunction. MnSOD has garnered attention for its pivotal role in preserving mitochondrial integrity under oxidative stress. This review aims to provide an updated overview of MnSOD’s structure, function, and relevance to human disease, offering insight into its therapeutic potential.

## 2. MnSOD

MnSOD is exclusively located within the mitochondrial matrix and is the only SOD within the mitochondria [8,9]. MnSOD defends against oxidative stress by converting O_2_^•−^ into H_2_O_2_ and oxygen. In 1969, the enzymatic activity of SODs was discovered when a copper protein was identified that could catalytically remove the Pauling free radical, the first experimentally recognized form of superoxide [10,11]. This discovery marked the first clear evidence that superoxide was a biologically relevant free radical and led to the identification of SODs as essential antioxidant enzymes. MnSOD was first isolated in E. coli in 1970 and was initially thought to be a homodimer of approximately 40 kDa, with each subunit weighing approximately 20 kDa [12]. In 1972, mitochondrial MnSOD was isolated from chicken liver with a calculated molecular weight of 79,400 (twice that of bacterial SOD), comprising four identical subunits, each weighing around 20,200 kDa, making it a homotetramer. The human MnSOD, encoded by MnSOD on chromosome 6q25.3, has a highly conserved sequence with over 40% homology shared among the human, yeast, and E. coli proteins [13,14]. The function of this homotetramer is essential for normal cellular metabolism, and its deficiency or dysfunction is linked to various diseases, including neurodegenerative, kidney, and cardiovascular conditions [7,15,16,17]. MnSOD is regulated through transcriptional, epigenetic, and post-translational modifications to ensure appropriate activity in response to oxidative stress and environmental factors [17,18].

### 2.1. Structure and Mechanism of Action

MnSOD operates as a tetrameric enzyme, composed of four identical subunits, each housing a catalytically essential manganese ion (Mn^2+^/Mn^3+^) within its active site. This tetrameric organization enhances structural stability and promotes intersubunit interactions that are key to the enzymatic efficiency of MnSOD [19,20]. This structural feature is exemplified in S. cerevisiae MnSOD, which shows heightened resistance to thermal and chemical unfolding compared to the dimeric C. albicans form, which more readily dissociates when crucial interfacial residues are altered [21]. In human MnSOD, residues glutamic acid (Glu)162 and histidine (His)30 are crucial at the dimer interface, where they form hydrogen bonds that help maintain structural integrity and catalytic competence [22,23]. Substitution of Glu162 has been shown to reduce MnSOD’s enzymatic activity by as much as 95% [24].

**Figure 2:**
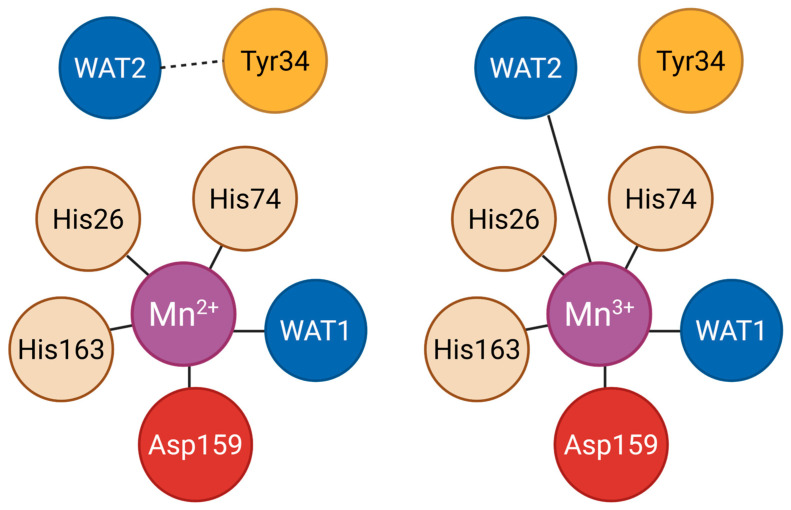
Coordination environment of Mn^2+^ and Mn^3+^ in human MnSOD. Comparison of the MnSOD active site in its reduced (Mn^2+^) and oxidized (Mn^3+^) states. Mn^2+^ is coordinated by His26, His74, His163, Asp159, and WAT1, with WAT2 hydrogen-bonded to Tyr34. In the Mn^3+^ state, WAT2 directly coordinates the metal center, replacing the Tyr34 interaction to complete the six-ligand geometry.

At the catalytic core, the manganese ion is ligated by inner sphere residues His26, His74, His163, and aspartic acid (Asp)159, along with a coordinating solvent molecule (referred to as WAT1, the first-shell water ligand bound to the manganese ion) that may exist as water or hydroxide [23]. The coordination geometry of the Mn^2+^ and Mn^3+^ active sites is illustrated in Figure 2. This configuration is essential for SOD [25]. Supporting this core, outer sphere residues including His30, tyrosine (Tyr)34, phenylalanine (Phe)77, tryptophan (Trp)78, Trp123, glutamine (Gln)143, Trp161, and Glu162 form a hydrogen bond network that facilitates proton transfer and stabilizes the catalytic transition states [22,23].

McAdam et al. described a four-step catalytic cycle for MnSOD consisting of a fast outer sphere pathway and a slower inner sphere route (Table 1) [26]. In both pathways, the cycle begins with the reduction of Mn^3+^ to Mn^2+^ by the first O_2_^•−^, accompanied by protonation of the coordinated hydroxide by Gln143 to form water (Step 1, rate constant k_1_) [26,27]. In the fast pathway, a second O_2_^•−^ oxidizes Mn^2+^ back to Mn^3+^ while generating H_2_O_2_ via interaction with Tyr34 and WAT1 (Step 2, k_2_) [26,28]. Under conditions of high O_2_^•−^ or low temperature, a reversible peroxide adduct forms with Mn^2+^ (Step 3, k_3_), temporarily inhibiting enzyme activity. The resolution of this product-inhibited complex requires two protons, delivered by WAT1 and surrounding outer sphere residues, to regenerate Mn^3+^ and release H_2_O_2_ (Step 4, k_4_) [20]. The ratio of k_2_/k_3_, referred to as the gating ratio, determines which pathway predominates [26].

Azadmanesh et al. later proposed a detailed mechanism centered on concerted proton-electron transfer (CPET), in which WAT1 and residues such as His26, His74, His163, Asp159, and Gln143 mediate coupled redox transitions of the manganese ion [25]. In the Mn^3+^ state, WAT1 is deprotonated and hydrogen-bonded to Gln143 and Asp159. Upon reduction to Mn^2+^, WAT1 is reprotonated via Gln143, which stabilizes the reduced state by forming strong hydrogen bonds with Trp123 and WAT1. A sixth ligand, possibly a hydroxide ion, also participates in stabilizing the Mn^2+^ conformation and facilitating the next catalytic round.

Recently, direct ultrafast crystallographic snapshots provided experimental confirmation of this CPET model. The study captured, at microsecond resolution, the real-time proton transfer from Gln143 to WAT1 and conformational shifts involving Trp123 as Mn transitioned between oxidation states. These findings validate the dynamic proton relay system involving Gln143–WAT1–Trp123 that drives MnSOD catalysis [29].

Temperature plays a crucial role in modulating these steps by influencing the gating ratio. While the initial steps (k_1_, k_2_) are relatively temperature-insensitive, the rates of peroxide adduct formation and its resolution (k_3_, k_4_) increase with temperature, peaking around 55 °C [26]. This shift favors the slower, inhibited cycle. Conversely, lower temperatures reduce enzyme inhibition, maintaining rapid turnover via the fast pathway [30].

### 2.2. Transcriptional and Post-Translational Regulation of MnSOD

**Figure 3:**
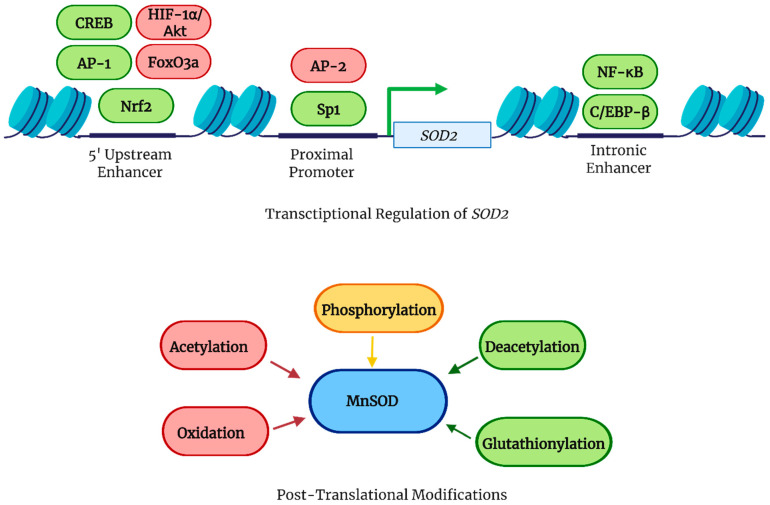
Regulation of MnSOD transcription and activity.

Transcriptional regulation and post-translational modifications can modulate the activity and stability of the enzyme. Three main regions of SOD2 are involved in regulating MnSOD expression: the 5′ upstream enhancer, the proximal promoter, and the intronic enhancer [31]. The 5′ upstream enhancer region contains binding sites for many transcription factors, namely activator protein-1 (AP-1), cAMP response element-binding protein (CREB), nuclear factor erythroid 2-related factor 2 (Nrf2), forkhead box O3a (FoxO3a), nuclear factor kappa-light-chain-enhancer of activated B cells (NF-κB), and hypoxia-inducible factor 1-alpha (HIF-1α) [16]. Nrf2 binds to the antioxidant response element in the promoter region of antioxidant genes, consequently increasing MnSOD expression; similarly, PGC-1α promotes the expression of antioxidative enzymes, including MnSOD [16]. Additionally, PGC-1α induces Nrf2-mediated MnSOD expression in the liver during Staphylococcus aureus-induced peritonitis [32]. HIF-1α and protein kinase B (Akt)-induced phosphorylation of forkhead box O3a (FoxO3a) downregulates MnSOD transcription, whereas sirtuin 3 (SIRT3), a mitochondrial nicotinamide adenine dinucleotide (NAD^+^)-dependent deacetylase, deacetylates FoxO3a and has the opposite effect [16]. The proximal promoter of MnSOD has CG repeats with binding sites for specificity protein 1 (Sp1), a transcriptional activator that promotes MnSOD expression. Activating protein-2 (AP-2), a transcription factor, also binds to the proximal promoter and competes with Sp1 for binding to the same promoter regions. When AP-2 binds, it can either directly inhibit Sp1 by occupying these sites or form AP-2/Sp1 complexes. The formation of AP-2/Sp1 complexes sequesters Sp1 away from the promoter, preventing it from activating MnSOD transcription effectively, thus reducing MnSOD expression [31]. Lastly, the intronic enhancer, located within the second intron, enhances Sp1 activity in the proximal promoter and possesses a nuclear factor kappa-light-chain-enhancer of activated B cells (NF-κB) binding site that interacts with CCAAT/enhancer-binding protein beta (C/EBP-β) to promote MnSOD transcription. Additionally, MnSOD is susceptible to inactivation by peroxynitrite (ONOO^−^), which nitrates critical tyrosine residues [16,31,33,34]. These regulatory mechanisms are summarized in Figure 3.

Post-translational modifications of MnSOD include acetylation, nitration, phosphorylation, and glutathionylation. The acetylation of human MnSOD at lysine residues is regulated by SIRT3 [35,36]. Acetylation reduces SOD activity, resulting in higher mitochondrial O_2_^•−^ levels, whereas irradiation, nutrient scarcity, and oxidative stress trigger SIRT3-mediated deacetylation of MnSOD to adequately enhance ROS scavenging within mitochondria to combat the emerging challenging conditions [16]. Nitric oxide is an intercellular messenger that has the potential to form ONOO^−^, which can inactivate MnSOD by nitrating Tyr34 at the active site [37]. Phosphorylation and dephosphorylation can either increase or decrease MnSOD activity depending on the specific amino acid residue modified, demonstrating a target site-based effect [16]. Like many other enzymes, permanent oxidation has the ability to pathologically shut down the activity of MnSOD, and S-glutathione, due to its ability to protect thiols from irreversible oxidation, can modulate MnSOD activity [16]. These post-translational modifications are also included in the schematic overview presented in Figure 3.

### 2.3. MnSOD as a Thermoreceptor

A recently proposed function of MnSOD is as a catalytic thermoreceptor. At lower temperatures, the enzyme increasingly favors the fast catalytic cycle, and when the temperature drops sufficiently, all reactions shift exclusively to this pathway, resulting in a marked increase in enzymatic activity [30]. This shift defines a threshold below which MnSOD becomes specifically activated by cold. For example, at 25 °C, the gating ratio of human MnSOD is approximately 1, while at 20 °C, it rises to 4, indicating a strong preference for the fast cycle under cold conditions [38].

Cold activation of MnSOD represents a proactive cellular adaptation to low temperatures, as the enzyme’s activity is upregulated beyond what is required to manage baseline superoxide levels. Kinetic modeling indicates that lower mitochondrial superoxide concentrations can drive the coenzyme Q reaction forward, increasing electron leakage and thereby amplifying H_2_O_2_ production by MnSOD [30,39]. MnSOD plays a central role in balancing superoxide and H_2_O_2_ fluxes to preserve redox homeostasis, a function that becomes especially crucial during temperature fluctuations. Under cold conditions, MnSOD not only accelerates SOD reactions via the fast cycle but also contributes to temperature regulation by promoting thermogenesis and ATP production, enabling efficient heat and energy generation. In contrast, higher temperatures favor the slower, product-inhibited cycle, potentially resulting in superoxide accumulation. By shifting toward the fast cycle at low temperatures, MnSOD enhances ROS detoxification and the thermogenic conversion of nicotinamide adenine dinucleotide (NADH) and flavin adenine dinucleotide (FADH_2_), highlighting its essential role in maintaining cellular energy and temperature balance during adaptive thermogenesis [30]. Excess H_2_O_2_ from cold-activated MnSOD may signal low temperatures and trigger physiological responses [30]. H_2_O_2_, a product of MnSOD activity, acts as a signaling molecule in energy metabolism and thermogenesis, modulating the uncoupling proteins by desensitizing them to inhibition and activating them through modifications like sulfenylation of UCP1-Cys253 and glutathionylation of UCP2-3 [40]. Additionally, H_2_O_2_ activates mitochondrial phospholipase A2γ, releasing free fatty acids that promote superoxide-induced uncoupling and increase cellular respiration; glutathione counters these effects [41,42].

The interaction between H_2_O_2_ and glutathione highlights how redox balance plays an important role in thermogenesis. In mammals, brown adipose tissue produces heat using the uncoupling proteins that are regulated by temperature and norepinephrine signals [40]. Some evidence suggests that transient receptor potential channels help sense temperature and may be influenced by ROS, although the details are still unclear [43,44]. When MnSOD is deleted, superoxide levels increase while H_2_O_2_ decreases, which impairs the body’s ability to generate heat. This finding supports the idea that both MnSOD and H_2_O_2_ are important for proper thermogenic responses [30]. Because MnSOD is found in all cells and responds to temperature shifts, it has been suggested that it might act as a general thermosensor across different tissues [30].

### 2.4. MnSOD and Obesity

MnSOD is essential for regulating mitochondrial function and energy metabolism in adipocytes, and its dysfunction has far-reaching consequences for obesity and metabolic health. Alterations in MnSOD expression or activity, whether genetic, diet-induced, or tissue-specific, can influence adiposity, insulin sensitivity, and oxidative stress. In adipocyte-specific SOD2 knockout mice that were fed a high-fat diet, MnSOD deletion surprisingly led to reduced fat mass, smaller adipocytes, lower leptin levels, and improved glucose and insulin tolerance, despite elevated superoxide levels and no major upregulation of compensatory antioxidant enzymes [45]. These mice also showed enhanced fatty acid oxidation, increased mitochondrial biogenesis, and protection from hepatic steatosis and inflammation, suggesting that adipocyte MnSOD normally supports energy storage and that its absence may favor energy expenditure through mitochondrial uncoupling.

Conversely, systemic MnSOD impairment appears to promote obesity. A study of 815 elderly individuals found that the Val/Val genotype of the Ala16Val MnSOD polymorphism—known to impair mitochondrial import of MnSOD—was more common among obese participants, highlighting how genetic disruption of mitochondrial antioxidant defenses may predispose individuals to oxidative stress and fat accumulation, thereby supporting a link between oxidative imbalance and increased adiposity [46]. Similarly, mice lacking MnSOD in intestinal epithelial cells developed spontaneous obesity marked by elevated inflammation, increased lipogenesis, and insulin resistance. These effects were driven by phospholipase A2 activation and excessive arachidonic acid release, but were reversed by an essential fatty acid-deficient diet [47]. A negative correlation between intestinal MnSOD and obesity markers in human samples further supported its translational relevance.

In another setting, skeletal muscle biopsies from obese pregnant women, with and without gestational diabetes, revealed reduced MnSOD and complex II (succinate dehydrogenase) activity compared to normal-weight controls, despite unchanged MnSOD protein levels [48]. Complex II is a key enzyme in both the tricarboxylic acid (TCA) cycle and the electron transport chain, and its impaired activity reflects mitochondrial dysfunction. The diminished activity was linked to increased acetylation of MnSOD due to reduced SIRT3 expression and activity, implicating impaired deacetylation as a mechanism for reduced antioxidant function. These women also exhibited greater oxidative stress, decreased PGC-1α expression, and a compromised mitochondrial capacity, reinforcing the notion that obesity itself can suppress MnSOD function.

Altogether, these studies reveal a nuanced relationship between MnSOD and obesity: while targeted loss of MnSOD in adipocytes may promote leanness and metabolic benefits, systemic or nonadipose MnSOD deficiency tends to drive inflammation, insulin resistance, and fat accumulation. Moreover, obesity may itself reduce MnSOD function, creating a feedback loop that worsens oxidative stress and mitochondrial dysfunction. These findings highlight the potential of MnSOD and its regulatory pathways as therapeutic targets for obesity-related metabolic disorders, though tissue-specific strategies will be crucial moving forward.

### 2.5. MnSOD and Renal Disease

The kidney is a metabolically demanding organ with high mitochondrial oxidative activity, particularly within the proximal tubular cells responsible for nutrient reabsorption after glomerular filtration. This makes it especially susceptible to oxidative stress-induced damage [16]. In particular, diabetes—a leading cause of chronic kidney disease—is closely associated with mitochondrial oxidative stress and MnSOD dysfunction. Mitochondrial oxidative stress has been directly implicated in the development and progression of chronic kidney disease and acute kidney injury, as elevated mitochondrial ROS can drive mitochondrial dysfunction, ATP depletion, inflammation, and apoptosis [16,49,50]. MnSOD plays a protective role in maintaining renal health. MnSOD-deficient mice show elevated oxidative stress, tubular injury, glomerular sclerosis, and increased infiltration of renal interstitial T cells and macrophages compared to wild-type controls [51]. In ischemia/reperfusion acute kidney injury models, oxidative stress leads to MnSOD nitration and deactivation, contributing to mitochondrial damage [52,53]. Even when kidney-specific MnSOD knockout mice maintain overall renal function, localized damage occurs, underscoring MnSOD’s importance in sustaining redox balance in renal mitochondria [54]. In albumin-overload models, decreased MnSOD levels are associated with increased mitochondrial oxidative stress and renal inflammation. In chronic renal allograft rejection and diabetic kidney disease, MnSOD dysfunction correlates with disease progression and elevated oxidative stress, and several therapeutic interventions targeting these pathways have shown promise [55,56,57]. Similarly, in membranous nephropathy, Moshen granule treatment alleviated kidney injury by activating the aryl hydrocarbon receptor (AhR), which upregulated Nrf2 and subsequently enhanced MnSOD expression, reducing oxidative stress and inflammation [58]. In a more recent model, proximal tubule-specific deletion of MnSOD in diabetic mice produced stage-dependent effects: early diabetes was associated with improved kidney function via NRF2 activation and enhanced fatty acid oxidation, while late-stage disease showed worsened renal injury due to impaired mitochondrial respiration and heightened inflammation, highlighting MnSOD’s dual role in disease progression [59]. Genetic studies have also linked the Ala16Val polymorphism in the MnSOD gene to diabetic kidney disease [60]. The Val/Val genotype is associated with increased diabetic nephropathy risk in Finnish, Swedish, and Japanese patients, while the Ala/Ala and Ala/Val genotypes are protective in Chinese and Mexican populations [61,62,63,64,65,66,67]. These genotypes differ in how efficiently MnSOD is imported into the mitochondria: the Val variant impairs import by altering the targeting sequence’s secondary structure, reducing mitochondrial MnSOD levels and antioxidant protection, whereas the Ala variant facilitates efficient import and preserves mitochondrial defenses [68].

MnSOD is also deeply involved in diabetes pathology. Type 1 and type 2 diabetes mellitus (T1DM and T2DM, respectively) are worsened by oxidative stress driven by glucose autoxidation [69,70,71]. The CT genotype of the MnSOD 47C/T polymorphism is associated with reduced MnSOD mRNA levels, which in turn increases susceptibility to type 1 diabetes mellitus (T1DM) in newly diagnosed patients [72,73]. In streptozotocin (STZ)-induced T1DM mice, MnSOD overexpression in pancreatic β-cells improved glucose control by inhibiting NF-κB activation and scavenging ROS [74]. Similarly, islets overexpressing MnSOD isolated from non-obese diabetic mice during the prediabetic stage and transplanted into STZ-induced diabetic severe combined immunodeficiency mice resulted in improved survival and prolonged function [75]. Long-term hyperglycemia causes diabetic complications, including nephropathy, neuropathy, and retinopathy, largely through oxidative stress-induced β-cell dysfunction and insulin resistance [70,76]. In diabetic cardiomyopathy, MnSOD overexpression preserves mitochondrial function and contractility by enhancing catalase activity and reducing oxidative damage [77,78]. MnSOD also protects against oxidative stress-induced apoptosis in detrusor smooth muscle during diabetic bladder dysfunction [79] and prevents diabetic retinopathy by reducing glucose-induced mitochondrial DNA damage [80]. Consistent with this, the multifunctional redox-sensitive protein DJ-1 (also known as PARK7) was shown to protect retinal ganglion cells from high glucose-induced oxidative stress by restoring MnSOD expression and activity, thereby maintaining mitochondrial membrane potential and promoting neuronal survival [81].

Recently, the mitochondrial acetyltransferase GCN5L1 was found to be upregulated in diabetic kidney disease, leading to increased MnSOD acetylation and decreased activity, exacerbating oxidative stress and renal injury [82]. Knocking down GCN5L1 restored MnSOD activity, reduced oxidative stress, and improved renal inflammation and fibrosis, suggesting that this pathway could be a therapeutic target [82].

In summary, the kidney’s metabolic intensity makes it vulnerable to mitochondrial oxidative damage; here, MnSOD serves a protective role by preserving redox balance and mitochondrial function [16,51,54]. Its dysfunction is implicated in multiple kidney diseases, including membranous nephropathy and diabetic nephropathy, in which both genetic polymorphisms (such as Ala16Val and 47C/T) and disease stage influence the disease outcomes [58,59,60,72]. MnSOD also protects against a wide range of diabetes-related complications, including cardiomyopathy, retinopathy, and transplant rejection, and is supported by additional regulators such as DJ-1 and GCN5L1, which modulate MnSOD activity under oxidative stress [81,82]. Targeting MnSOD function—whether by enhancing its expression, preventing its inactivation, or regulating upstream effectors—may offer promising therapeutic strategies for both renal and metabolic diseases [82].

### 2.6. MnSOD in Cardiovascular Disease

The heart is one of the most energy-demanding organs in the body and consequently generates high levels of ROS [83]. This makes it particularly sensitive to impairments in antioxidant defense systems, especially those involving MnSOD. The role of MnSOD in the cardiovascular system is complex, as high and low levels can impact the disease risk differently depending on the tissue and cell type involved. For this reason, MnSOD expression and activity levels have been investigated as predictive biomarkers for cardiovascular conditions, even when those conditions are not directly caused by MnSOD dysfunction [83]. In some cases, elevated MnSOD is protective, while in others, it may reflect an adaptive response to pathology and correlate with worse outcomes [84].

Oxidative stress is a key factor in hypertension, a disease where MnSOD plays a protective role. Oxidative stress contributes to vascular stiffness and smooth muscle cell adhesion, exacerbating endothelial dysfunction and increasing blood pressure [85,86]. Within the brain, MnSOD regulates blood pressure by reducing mitochondrial superoxide anions. Deletion of MnSOD in the subfornical organ raises the mean arterial pressure and intensifies angiotensin II-induced hypertension in mice [87]. Similarly, reduced MnSOD in the brainstem rostral ventrolateral medulla of hypertensive rats leads to elevated ROS and blood pressure, whereas MnSOD gene delivery to this region lowers arterial pressure [88]. Delivering MnSOD to carotid and femoral arteries also mitigates hypertension and delays vascular dysfunction [89].

Findings in neonatal pulmonary hypertension have also implicated MnSOD. Loss of MnSOD function in animal models leads to persistent pulmonary hypertension of the newborn (PPHN), with elevated right ventricular pressure and endothelial apoptosis [90]. Reintroducing MnSOD restores nitric oxide signaling and improves vascular relaxation, indicating therapeutic promise for this approach in PPHN [90,91].

Genetic variations in MnSOD influence the susceptibility to cardiovascular diseases. In a Taiwanese population, the Val/Ala or Ala/Ala MnSOD genotypes were associated with increased coronary artery disease risk and greater severity in patients with one- or two-vessel disease [92]. No such associations were observed for catalase or glutathione peroxidase polymorphisms, making MnSOD an independent risk factor [92]. These results were mirrored in a Tunisian cohort [93].

Cardiomyocyte-specific SOD2 knockout in mice results in early-onset dilated cardiomyopathy and death by four months due to heart failure [92,94]. The mice displayed mitochondrial abnormalities, excessive ROS, impaired respiration, and metabolic shifts without compensatory antioxidant responses [92]. A related inducible model with SOD2+/− and SOD2−/− mice found that global knockout led to early mortality and reduced activity, while heterozygous deletion impaired cardiac function, promoted fibrosis, and heightened sensitivity to doxorubicin-induced oxidative stress [95].

Other cardiovascular structures, such as aortic valves, are also affected by MnSOD levels. Mice with MnSOD haploinsufficiency showed increased oxidative stress and calcification without functional valve changes, whereas MnSOD overexpression enhanced osteogenic markers without lowering the calcium burden, highlighting complex effects depending on the disease stage and cell type [96].

The link between MnSOD and atherosclerosis is well documented. Loss of MnSOD in ApoE-deficient mice accelerates plaque formation, enlarges necrotic cores, and promotes vascular inflammation, especially at arterial branch points prone to high shear stress [97,98,99].

Therapeutically, MnSOD also appears to mediate the protective effects of tamoxifen. In cardiomyocytes, tamoxifen increased MnSOD levels and reduced apoptosis following adriamycin exposure, suggesting that MnSOD induction is one mechanism for its cardioprotective effects [100].

Mitochondrial oxidative stress plays a major role in diabetic cardiomyopathy, where MnSOD overexpression has shown protective effects. In T1DM mice, cardiac-specific SOD2 overexpression preserved mitochondrial structure, restored glutathione levels, and maintained cardiac function, although full respiratory function was not completely restored [78].

Platelets are also affected by MnSOD. In its absence, platelet mitochondria produce excess ROS, which increase platelet activation and aggregation, promoting thrombosis and contributing to cardiovascular disease risk [101]. These effects underscore MnSOD’s broader role in vascular health and inflammation [101]. Supporting this, a reduction in mitochondrial ROS through SOD2-targeted therapy improved oxidative phosphorylation and preserved endothelial resilience in non-reperfused myocardial infarction, highlighting MnSOD’s therapeutic potential in protecting coronary endothelium under ischemic conditions [102].

Atrial fibrillation (AF) may also be linked to MnSOD levels. A study measuring plasma MnSOD in 130 patients found the highest levels in those with paroxysmal AF, with lower levels in persistent AF and the lowest levels in the controls [103]. These levels correlated with atrial size and red blood cell metrics, and logistic regression identified MnSOD as an independent risk factor for paroxysmal AF [103]. However, whether this reflects a compensatory response or causal involvement remains unclear.

When it comes to heart failure prognosis, elevated MnSOD levels have been linked to poor outcomes. In one cohort, the patients who died or required transplant had higher MnSOD activity, which negatively correlated with ejection fraction and renal function while positively correlating with N-terminal pro b-type natriuretic peptide (NT-proBNP) levels [84]. This may indicate an adaptive MnSOD response to cardiac stress rather than direct pathogenic involvement. Recent findings showed that heat stress downregulates MnSOD expression in endothelial cells via p53-mediated disruption of Sp1 binding to the MnSOD promoter, resulting in oxidative damage; this suppression was rescued by treatment with the mitochondria-targeted antioxidant MitoQ10 or the p53 inhibitor Pifithrin-α, highlighting potential therapeutic strategies to preserve vascular redox balance under stress conditions [104].

Altogether, MnSOD is essential for cardiovascular health, mitigating oxidative stress in the heart, blood vessels, and circulating cells [83,85,86]. Its deficiency has been linked to hypertension, atherosclerosis, diabetic heart disease, and cardiomyopathy, while its overexpression or adaptive increases can signal either protection or underlying damage depending on the context [84,92,94,96]. Understanding the tissue-specific and disease-dependent roles of MnSOD could help optimize therapeutic strategies, especially those involving MnSOD mimetics, targeted antioxidants like MitoQ10, or gene therapy approaches to modulate oxidative stress [78,100,101,104].

### 2.7. MnSOD in Cancer

MnSOD plays a multifaceted role in cancer, exerting both tumor-suppressive and tumor-promoting effects depending on the context. As a key mitochondrial antioxidant enzyme, it protects critical components of cellular metabolism, including enzymes in the TCA cycle, from oxidative damage—a function essential for maintaining metabolic integrity and preventing malignant transformation [105,106,107,108,109]. Loss of MnSOD has been shown to enhance tumor growth, invasiveness, and tumorigenicity, while its reintroduction suppresses these features in multiple xenograft models [110,111,112,113,114,115]. Paradoxically, elevated MnSOD expression is sometimes observed in aggressive cancers, where it contributes to resistance to chemotherapy and radiotherapy [7,116,117,118,119,120].

This context-dependent behavior has been demonstrated in clinical data. In diabetic patients with endometrial cancer, high MnSOD levels are linked to worse overall survival, although this effect can be mitigated by statin therapy [121]. Similarly, in colon adenocarcinoma, increased MnSOD expression in adjacent normal tissues—but not within tumors themselves—correlates with poor prognosis, further supporting its role as a nuanced prognostic indicator [122]. In pancreatic cancer, MnSOD is upregulated via peroxisome proliferator-activated receptor gamma (PPARγ), which promotes tumor proliferation by suppressing autophagy-related protein 4D (ATG4D)-mediated mitophagy, thus preventing mitochondrial reactive oxygen species (ROS)-induced apoptosis [123]. This reflects a mechanistic switch whereby MnSOD supports tumor survival under oxidative stress by inhibiting cell death pathways.

MnSOD is encoded by the superoxide dismutase 2 (SOD2) gene on chromosome 6q25.3. While this region is generally stable in cancer, some cases—such as melanoma—exhibit loss of heterozygosity or decreased expression in virally transformed fibroblasts [124,125,126]. Reduced expression often arises from epigenetic silencing, including promoter hypermethylation and chromatin remodeling, whereas overexpression tends to result from transcriptional activation by oxidative stress or inflammatory signaling [124]. Transcription factors such as specificity protein 1 (Sp1) and AP-2 regulate SOD2 transcription, and silencing of AP-2 by hypermethylation has been linked to MnSOD upregulation in aggressive breast cancer [34,127,128]. Mutations in the promoter region that impair AP-2 binding or interfere with Sp1 recruitment further reduce MnSOD transcription [129,130]. These alterations are commonly observed in breast, multiple myeloma, and pancreatic cancer cell lines [131,132,133,134].

The tumor suppressor TP53, encoding the p53 protein, also modulates MnSOD levels. High p53 activity represses MnSOD, while low expression or mutated p53 allows for activation of nuclear factor kappa-light-chain-enhancer of activated B cells (NF-κB)-driven transcription [110,135,136,137,138,139]. Notably, MnSOD can physically interact with p53 within mitochondria to initiate apoptosis [140]. This dual regulatory role is evident in gastric cancer, where exogenous thermostable MnSOD inhibits tumor progression, upregulates p53, and downregulates zinc finger E-box-binding homeobox 1, implicating MnSOD in both transcriptional and redox-based tumor suppression [141].

MnSOD transcription is also regulated by stress response pathways. NF-κB binds an intronic enhancer in SOD2, promoting transcriptional activation in metastatic breast cancer [33,34,142], thereby enhancing survival and resistance to apoptosis [143,144]. Tumor necrosis factor alpha-induced NF-κB activation also boosts MnSOD expression to counter ROS toxicity [145]. Similarly, Nrf2 upregulates MnSOD by binding the antioxidant response element upon kelch-like ECH-associated protein 1 (Keap1) inhibition [146,147,148,149,150]. Nrf2 activation or Keap1 mutations can contribute to chemoresistance and MnSOD overexpression [151,152,153,154].

Forkhead box O3a (FoxO3a), another key transcription factor, is modulated by the phosphoinositide 3-kinase (PI3K)/protein kinase B (Akt) pathway. Akt phosphorylates FoxO3a, excluding it from the nucleus and suppressing MnSOD expression [155,156,157]. Sirtuin 3 (SIRT3) enhances FoxO3a transcriptional activity via deacetylation, promoting MnSOD expression during oxidative stress [158]. These interactions are functionally important in glioma, where 6-gingerol induces apoptosis via MnSOD and extracellular signal-regulated kinase (ERK) activation. Knockdown of MnSOD or inhibition of ERK abolishes these effects, demonstrating MnSOD’s central role in stress-mediated cell death [159]. Dimethyl fumarate (DMF) also suppresses tumor progression in oral squamous carcinoma by upregulating MnSOD, reducing oxidative stress, and reversing epithelial–mesenchymal transition [160]. Likewise, a triple drug combination—metformin, efavirenz, and fluoxetine—induces ROS overload and upregulates MnSOD, overwhelming antioxidant defenses and causing selective cancer cell apoptosis [161].

Additional regulators include aryl hydrocarbon receptor nuclear translocator in acute myeloid leukemia (AML), CREB-1, activating transcription factor 1 (ATF-1), and HIF-1α [162,163]. Stem cell-associated factors Nanog and Oct4 can also control MnSOD transcription [164,165,166,167].

Post-transcriptionally, MnSOD is suppressed by microRNAs (miRNAs) targeting its 3′ untranslated region. An Alu-like element within the 3′UTR recruits small RNAs that block translation [168]. For instance, miR-222 and miR-382 downregulate MnSOD in tongue cancer and under transforming growth factor beta signaling [169,170], while low miR-17 and miR-146a levels are associated with increased MnSOD and altered treatment responses in prostate and ovarian cancer [171,172,173].

MnSOD function is also modulated by genetic and post-translational changes. Single-nucleotide polymorphisms like Ile58Thr, Leu60Phe, and Ala16Val reduce activity and have been linked to cancer susceptibility [8,68,174,175,176,177,178,179,180]. In gastric cancer, Val/Val genotypes are enriched, supporting its role as a risk factor [181]. Post-translational modifications such as acetylation, phosphorylation, oxidation, nitration, and ubiquitination affect MnSOD function [124]. Notably, acetylation at lysine 68 (K68) converts MnSOD from an antioxidant tetramer to a pro-oxidant monomer, promoting tumor growth [182]. K68-acetylated MnSOD confers resistance to chemotherapy and enhances the tumorigenic potential, while also promoting stemness through HIF-2α stabilization and activation of Oct4, Sox2, and Nanog [182,183].

MnSOD is deeply integrated into cancer metabolism. It supports the Warburg effect via AMP-activated protein kinase (AMPK) and glucose transporter 1 activation [184,185,186,187,188,189,190]. In gastric cancer, toll-like receptor 2 induces MnSOD and glycolysis, suggesting that MnSOD has potential as a biomarker [191]. MnSOD also supports mitochondrial respiration under nutrient stress and promotes proteasomal degradation, thereby enhancing cancer cell survival during metabolic challenges [192].

Lastly, MnSOD modulates the tumor immune microenvironment. It is associated with C-X-C motif chemokine ligand 8 (CXCL8)-mediated neutrophil infiltration [193], CD68-positive macrophages in lung cancer [194], and M2 macrophage polarization in triple-negative breast cancer [195]. Notably, MnSOD-overexpressing oncolytic viruses recruit lymphocytes and enhance immunotherapy responses [196].

In summary, MnSOD shapes nearly every facet of tumor biology—from redox balance and metabolism to immune evasion and treatment resistance. Its dual roles, governed by diverse transcriptional, post-transcriptional, and post-translational mechanisms, highlight its potential as both a therapeutic target and diagnostic biomarker in oncology.

### 2.8. Implication in Neurodegenerative Diseases

Manganese superoxide dismutase (MnSOD) is primarily expressed in neurons throughout the brain and spinal cord, with lower basal levels in astrocytes; however, its expression in astrocytes increases significantly under conditions of oxidative stress [197]. Brain-specific SOD2-deficient transgenic mice exhibit perinatal death, a lower body weight, a reduced brain size, spongiform encephalopathy, and increased astrocyte activation in the cerebral cortex, brain stem, and hippocampus, alongside selective loss of mitochondrial complex II activity and elevated lipid peroxidation, consistent with oxidative stress-induced brain damage [197]. Epilepsy patients show increased reactive oxygen species (ROS) levels and decreased expression of antioxidant enzymes, including MnSOD [198]. Lower MnSOD expression is associated with greater oxidative damage and heightened neuronal excitability, both of which contribute to seizure susceptibility [198]. Preclinical models indicate that MnSOD mimetics alleviate oxidative stress and reduce seizure severity [199]. Clinical findings also suggest that apoptotic markers are elevated in epilepsy patients, particularly in those with the MnSOD Ala16Val polymorphism. This polymorphism is associated with increased interleukin-1β (IL-1β) and interleukin-6 (IL-6) levels, implicating MnSOD in inflammation-linked seizure pathology and highlighting the relevance of genetic variation in disease severity and immune responses [200]. Huntington’s disease is characterized by oxidative stress and mitochondrial dysfunction caused by mutant huntingtin, which disrupts mitochondrial respiration and elevates ROS levels. MnSOD helps attenuate ROS accumulation and protect neurons, with overexpression of MnSOD conferring neuroprotection in disease models [201,202]. In a 3-nitropropionic acid-induced model of Huntington’s disease, treatment with C_60_ fullerene reduced oxidative stress, improved mitochondrial function, and restored MnSOD levels, demonstrating its therapeutic potential through antioxidant pathway modulation [203].

Oxidative stress is also a major contributor to ischemic stroke, where MnSOD levels are often reduced in affected patients [204]. In transgenic mice, MnSOD overexpression or treatment with MnSOD mimetics mitigates ischemia/reperfusion injury and improves neurological outcomes [204,205]. Administration of irisin, a myokine linked to physical exercise, in a murine model of cerebral ischemia reduced mortality and improved cognition, effects that were associated with increased expression of MnSOD and the anti-aging protein Klotho, suggesting that MnSOD mediates the neuroprotective benefits of exercise-mimetic therapies in stroke [206]. Similarly, chronic low-dose alcohol consumption in a post-ischemic model attenuated inflammation via activation of peroxisome proliferator-activated receptor gamma (PPARγ), which correlated with increased MnSOD expression and reduced neuroinflammation [207]. Clinical data show that MnSOD levels inversely correlate with stroke severity, suggesting its potential as both a biomarker and therapeutic target [204,205]. In a rodent model of stroke, treatment with chlorpromazine and promethazine reduced infarct size and neuronal damage through modulation of the protein kinase C delta (PKC-δ)/nicotinamide adenine dinucleotide phosphate oxidase/MnSOD pathway, confirming MnSOD’s involvement in pharmacological neuroprotection during ischemic injury [208]. Notoginseng leaf triterpenes (NLTs), a compound derived from Panax notoginseng, protected against cerebral ischemia and oxygen-glucose deprivation/reoxygenation-induced neuronal injury by activating the nicotinamide phosphoribosyltransferase–sirtuin (SIRT)1/2/3–Forkhead box O3 (Foxo3a)–MnSOD signaling pathway, demonstrating MnSOD’s role as a key effector in NAD^+^-dependent antioxidant defenses [209,210].

In Parkinson’s disease (PD), oxidative stress contributes to dopaminergic neuron loss in the substantia nigra. Overexpression of MnSOD in transgenic mice improves survival following neurotoxin exposure, while MnSOD mimetics reduce ROS and neuroinflammation [211,212]. The mitochondrial deacetylase SIRT3 enhances MnSOD activity by preserving its active tetrameric structure through deacetylation, thereby reducing mitochondrial ROS and offering a neuroprotective mechanism in PD [213]. Additionally, inhibition of mixed-lineage kinase domain-like protein in a 1-methyl-4-phenyl-1,2,3,6-tetrahydropyridine-induced PD mouse model decreased oxidative stress and dopaminergic neuronal death, in part by increasing MnSOD expression, further implicating MnSOD in limiting PD-associated neurodegeneration [214]. Clinical studies have reported decreased MnSOD levels in patients, linking oxidative damage to disease progression [212].

In Alzheimer’s disease (AD), MnSOD plays a similar neuroprotective role. Oxidative stress and mitochondrial dysfunction are central features of AD, and MnSOD overexpression in transgenic mice reduces hippocampal superoxide levels and alleviates memory deficits [215,216]. In multiple AD models, modulation of MnSOD expression resulted in consistent neuroprotection, with reductions in ROS and improvements in cognitive function [216]. In a triple-transgenic AD (3×Tg-AD) mouse model with cerebral microinfarcts, activation of protein kinase C epsilon (PKCε) improved memory and hippocampal vascular integrity, effects associated with increased MnSOD expression, suggesting that MnSOD contributes to both neurovascular and cognitive protection in AD complicated by vascular injury [217]. In aged and AD hippocampi, PKCε activation also restored PKCε, MnSOD, and vascular endothelial growth factor levels and microvessel density, implicating MnSOD in a PKCε-regulated neurovascular repair axis that becomes dysregulated in aging and AD [218]. MnSOD is also significantly upregulated by DMF, a treatment used in neurodegeneration models, leading to improved mitochondrial function and redox balance. These effects are abrogated by silencing Nrf2, confirming MnSOD as a key downstream target of Nrf2-driven neuroprotection [219]. MnSOD expression is also increased via SIRT3 activation in amyloid-beta (Aβ)-treated models, where a novel rhamnoside derivative reduced ROS, restored mitochondrial integrity, and suppressed cellular senescence, reinforcing the MnSOD–SIRT3 axis as a promising neuroprotective pathway in AD [220]. Pramlintide, an amylin analog, enhanced cortical MnSOD expression and mitochondrial function while reducing oxidative damage in AD models, further validating MnSOD’s protective role [221]. In apolipoprotein E4 (ApoE4)-positive AD models, MnSOD expression in hippocampal microvessels was reduced, implicating MnSOD in vascular oxidative stress and suggesting a role for MnSOD in genetically predisposed cognitive decline [222]. Consistent with this, irradiated hippocampal slices from ApoE4 mice exhibited impaired ROS responses due to diminished MnSOD expression, underscoring MnSOD’s role in adaptive redox signaling under environmental and genetic stress [223].

Beyond classical neurodegenerative diseases, MnSOD also plays a protective role in neuroinflammatory disorders. In multiple sclerosis (MS), MnSOD expression is upregulated following ocrelizumab treatment, particularly in CD19^+^ B cells, suggesting a role in the drug’s neuroprotective and anti-inflammatory effects through PKCβ modulation [224]. Vitamin D supplementation in patients with relapsing–remitting MS also significantly increases MnSOD expression, supporting its function in broader antioxidant defenses during neuroinflammation [225]. Mice with neuron-specific MnSOD deletion exhibit demyelination, inflammation, and progressive paralysis, recapitulating the pathological features of progressive MS and demonstrating the importance of MnSOD in maintaining neuronal resilience against inflammation-induced damage [226]. Similarly, in experimental autoimmune encephalomyelitis, combination therapy with lovastatin and an AMPK activator improved mitochondrial and peroxisomal function, upregulated MnSOD, and reduced disease severity, positioning MnSOD as a therapeutic effector in metabolically guided treatment of MS [227].

In summary, MnSOD is vital for neuronal health throughout the central nervous system. It is constitutively expressed in neurons and becomes upregulated in astrocytes during oxidative stress [197]. Genetic deletion results in profound neurological deficits and early mortality [197], while reduced MnSOD expression is commonly observed in conditions such as epilepsy, Huntington’s disease, ischemic stroke, Parkinson’s disease, and Alzheimer’s disease [198,202,204,211,215]. In each of these disorders, therapeutic elevation of MnSOD levels—through genetic overexpression, pharmacologic mimetics, or upstream regulators like SIRT3 or Nrf2—consistently reduces the ROS burden and limits neuronal injury [199,201,205,212,216]. Therefore, MnSOD represents a promising target for managing oxidative stress for a broad spectrum of neurodegenerative and neuroinflammatory diseases.

## 3. Conclusions

MnSOD is a vital mitochondrial antioxidant enzyme whose structural stability, catalytic activity, and regulatory flexibility are central to maintaining cellular redox homeostasis. Its role in converting superoxide into H_2_O_2_ makes it essential for protecting mitochondrial function while also influencing broader metabolic and signaling pathways. MnSOD’s physiological importance spans thermogenesis, energy metabolism, and neuronal health, while its dysregulation is implicated in the development and progression of obesity, kidney disease, cardiovascular dysfunction, cancer, and neurodegeneration. Obesity impairs MnSOD expression and mitochondrial efficiency in brown adipose tissue and skeletal muscle, contributing to thermogenic failure and systemic metabolic dysfunction. Loss of MnSOD activity exacerbates lipid accumulation and insulin resistance, while its preservation supports mitochondrial respiration and energy expenditure. In the kidney, MnSOD dysfunction drives oxidative injury across multiple nephron compartments. Whether in acute kidney injury, chronic allograft rejection, or diabetic nephropathy, its loss results in elevated mitochondrial ROS, inflammation, and fibrosis. Restoration of MnSOD through genetic or pharmacologic means confers significant protection. The cardiovascular effects of MnSOD are highly context- and tissue-dependent: deficiency promotes hypertension, atherosclerosis, cardiomyopathy, and endothelial dysfunction, whereas upregulation may indicate either protective adaptation or pathological stress signaling. Within cancer biology, MnSOD assumes a dual role—suppressing tumor initiation by preserving mitochondrial integrity and limiting DNA damage, but later promoting tumor progression, angiogenesis, and therapy resistance through altered regulation and metabolic reprogramming. Finally, in neurodegenerative disorders, MnSOD deficiency heightens neuronal vulnerability to mitochondrial dysfunction and oxidative damage. This is particularly evident in Alzheimer’s, Parkinson’s, and diabetic retinopathy models, where its maintenance improves mitochondrial integrity, redox balance, and neuronal survival. These context-dependent effects are shaped by a network of regulatory mechanisms, including transcription factors, epigenetic marks, microRNAs, and post-translational changes that modulate MnSOD expression, localization, and activity. In several disease models, enhancing MnSOD levels or function, whether through genetic means, transcriptional activation, or targeted post-translational modulation, confers protective effects, while its deficiency exacerbates oxidative damage and dysfunction. To complement this, we have published a 2024 review of the current state of MnSOD mimetics, which have shown promise as pharmacological tools to replicate or enhance MnSOD activity in pathological settings [228]. Continued research into natural MnSOD regulation and the rational development of mimetics may yield effective strategies for managing oxidative stress-related diseases, particularly those in which direct genetic modulation is impractical. Together, these complementary approaches hold significant promise for advancing the treatment of a wide range of oxidative stress-driven conditions.

## Figures and Tables

**Table 1:** Stepwise mechanism of MnSOD catalysis: associated residues, pathways, and kinetics.

Step	Pathway	Reaction Description	Key Residues and Features	Rate Constant	References
1	Both	O_2_^•−^ reduces Mn^3+^ to Mn^2+^; hydroxide ligand is protonated to H_2_O by Gln143	Inner sphere: His26, His74, His163, Asp159, WAT1; Gln143 donates proton	k_1_	McAdam et al., 1977 [26]; Abreu et al., 2005 [27]
2	Fast	Second O_2_^•−^ oxidizes Mn^2+^ to Mn^3+^; produces H_2_O_2_ via reaction with Tyr34 and water ligand	Outer sphere: Tyr34, WAT1; enzyme is regenerated	k_2_	McAdam et al., 1977 [26]; Perry et al., 2009 [28]; Bonetta Valentino et al., 2022 [20]
3	Slow	Second O_2_^•−^ binds Mn^2+^ to form a reversible peroxide adduct that inhibits activity	Product-inhibited complex formation	k_3_	McAdam et al., 1977 [26]; Bonetta Valentino et al., 2022 [20]
4	Slow	Two protons regenerate Mn^3+^ and release H_2_O_2_, restoring activity	Proton donors: water ligand and outer sphere residues	k_4_	McAdam et al., 1977 [26]; Bonetta Valentino et al., 2022 [20]

## Abbreviations

The following abbreviations are used in this manuscript:

AD	Alzheimer’s disease
AF	Atrial fibrillation
AML	Acute myeloid leukemia
AMPK	AMP-activated protein kinase
AP-1 AP-2	Activator protein 1 Activator protein 2
ATF	Activating transcription factor
CPET	Concerted proton–electron transfer
CREB	cAMP response element-binding protein
DJ-1	the multifunctional redox-sensitive protein DJ-1 (PARK7)
DMF	Dimethyl fumarate
ERK	Extracellular signal-regulated kinase
HIF-1α	Hypoxia-inducible factor 1-alpha
IL	Interleukin
MS	Multiple sclerosis
NAD	Nicotinamide adenine dinucleotide
NADH	Reduced nicotinamide adenine dinucleotide
NF-κB	Nuclear factor kappa B
NRF2	Nuclear factor erythroid 2-related factor 2
ONOO	Peroxynitrite
PD	Parkinson’s disease
PGC-1α	Peroxisome proliferator-activated receptor gamma coactivator 1-alpha
PKC	Protein kinase C
PPHN	Persistent pulmonary hypertension of the newborn
ROS	Reactive oxygen species
SIRT3	Sirtuin 3
STZ	Streptozotocin
TCA	Tricarboxylic acid
WAT1	First-shell water ligand bound to manganese ion
